# Artificial intelligence and eating disorders: a commentary

**DOI:** 10.1007/s40519-023-01598-5

**Published:** 2023-08-05

**Authors:** Massimo Cuzzolaro

**Affiliations:** 1 Unitelma Sapienza University of Roma, Rome, Italy; 2 Medical Pathophysiology, Formerly Sapienza University of Roma, Via Fedi 12, 57021 Campiglia Marittima (LI), Rome, Italy

Great technological innovations always arouse both excitement and fear. It also happens with artificial intelligence (AI), which the Oxford English Dictionary defines as follows: “The capacity of computers or other machines to exhibit or simulate intelligent behavior; the field of study concerned with this” [1]. A few brief historical reminders may help to understand the current process.

The debate over the pros and cons of AI has become increasingly heated in recent years. Still, the premises go back to the middle of the previous century when Alan Turing devised the first electromechanical calculators. In 1950, in a famous seminal paper [2], Turing posed a key question, “Can machines think?”. Through the Turing test (also known as *the imitation game*), he sought to understand whether a human being asking questions through a terminal can distinguish answers written by another human being from those written by a computer.

The expression AI was coined a few years later when John McCarthy, a computer scientist from the United States, prepared with other mathematicians and cognitive scientists a conference destined to mark the birth date of a new discipline: Dartmouth Conference on AI, Hanover, New Hampshire, 1956. The goal was to study whether a machine can simulate every aspect of learning and every feature of human intelligence or whether there are unique, artificially irreproducible traits [3].

Soon a decisive step occurred. Frank Rosenblatt [4] devised a computer capable of learning through trial and error (deep learning). The process was entrusted to an artificial neural network inspired by how biological neurons activate and transmit signals. Artificial neural networks are the basis of AI.

Therefore, AI is the theory and development of automated information processing systems capable of choosing different options based on stored data and performing tasks that usually require human intelligence, such as recognizing a face, translating a text from one language to another, making a diagnosis, and playing chess.

Speaking of chess, 1996 world chess champion Garry Kasparov beat the Deep Blue computer. But a year later, Deep Blue, further developed, prevailed.

*Classic machine learning* refers to machines that use algorithms to analyze large amounts of data, learn from it, and produce informed decisions or predictions. However, these machines cannot generate new, original content like human creativity. In recent years, generative artificial intelligence (GAI) has been developed. The advent of GAI represented a further step forward. GAI systems learn not only to recognize data, but also to create new contents—texts, images, pieces of music—that resemble training data but are original and unique. Several GAI systems exist, such as Bard, ChatGPT, Dall-E, DeepMind, and Midjourney.

What effect will AI, particularly GAI, have in studying and treating eating disorders (EDs)?

The editorial “The Oracle of Delphi 2.0: Considering Artificial Intelligence as a Challenging Tool for the Treatment of Eating Disorders” [5] put together the result of an experiment and reflection on artificial intelligence (AI) and its possible applications.

The experiment consisted of asking ChatGPT (a well-known generative AI tool) to write an editorial on the use of AI for EDs. ChatGPT produced a 610-word, very rough text that contained only a few plausible suggestions. It became apparent that the tool must still be capable of writing a publishable scientific article. But progress is very rapid, and the problem will arise soon.

As to the study, prevention, and treatment of EDs, some possible uses and risks of AI are indicated by the editorial and some recent articles [5–11] (Table 1). They also apply to other fields of medicine [12].

**Table 1:** Artificial intelligence

Possible benefits	Possible risks
• Rapid processing of large amounts of data on both individuals and large samples • Early diagnosis (including through social media analysis, digital footprint monitoring) • Integrations via AI of psychotherapies conducted by specialists (e.g., AI-based support between treatment sessions) • Therapies conducted entirely by AI tools • …	• Non-transparent algorithms • Privacy violations • Dissemination of false information • In integrations, the patient may end up preferring the AI tool to the human relationship • Loss of the intersubjective relationship between human beings • Loss of the formations of the unconscious • Do-it-yourself treatments without control • …

We are entering a new era in the history of medicine. Over the past fifty years, the principles and laws that inspire and regulate the relationships of care and treatments placed at the service of people (in ancient Greek the word θεραπεία, service, indicated them) have undergone profound and precipitous changes. Two trend lines seem to parallel: enormous techno-scientific developments and progressive depersonalization of diseases and treatment relationships.

Now, more and more, GAI will help design research and discover new drugs. It will write clinical reports and scientific papers, read radiological examinations and electrocardiograms, make diagnoses, remotely monitor disease progress, communicate with patients, and continue to develop, hand in hand, robotic surgery.

One model of GAI—Generative Pre-trained Transformer 4 (GPT-4)—has produced correct diagnoses in several difficult cases with a high frequency, higher than that demonstrated by many physicians [13].

To take just one example in the field of eating and weight disorders, developments in functional magnetic resonance imaging combined with AI algorithms are an up-and-coming tool in the study of Prader–Willi syndrome, a rare and severe form of obesity—genetic and epigenetic—with uncontrolled eating and other compulsive symptoms [14].

Historian of medicine Pedro Laín Entralgo wrote that the Greek τεχνή ἰατρική required λόγος, φιλία, and ἔρως: knowledge of physiology and logical thinking, but also friendship for man and passion for the art of caring for him [15].

Will GAI lighten and improve clinical work? Will there be more space and time for the therapist–patient relationship? More attention to the subjectivity of both? Or will the efficiency of thinking machines—quick, tireless, emotionless, protected from forgetfulness and lapses, never at risk of burn-out—dominate the field? Will we be more capable of understanding and sharing the malaise and tragic moments of existence, or will we become more and more automatons in a world of robots, in societies of control and submission in which man risks becoming only a *machine à vivre*, according to an expression by Paul Valéry [16]?

As mentioned above, major technological innovations always arouse enthusiasm and fear. Appreciation of the benefits must be free from prejudices and open to the future. But it is equally necessary to remember that technical development and civil progress are not the same and do not necessarily go hand in hand. The uses and effects of inventions over long periods are largely unpredictable. But they depend, at least in part, on us.

Blaise Pascal (1623–1662)—mathematician, engineer, philosopher, and theologian—wrote: «il ne faut pas se méconnaître, nous sommes automate autant qu'esprit (fragment 661: we mustn't misunderstand ourselves, we are automatons as much as spirit) [17].

Machines are no better than humans who invent them according to the rules of *esprit de géométrie*. And human beings are no better than their machines if they do not keep alive what Pascal called *esprit de finesse*, which inspires the basic principles, the ethical and political values, and the rules of rules.

Those who will live will see.
